# The effect of various dilute administration of rocuronium bromide on both vascular pain and pharmacologic onset: a randomized controlled trial

**DOI:** 10.1186/s12871-019-0743-5

**Published:** 2019-05-15

**Authors:** Mayuko Kanazawa, Aiji Sato (Boku), Yoko Okumura, Mayumi Hashimoto, Naoko Tachi, Yushi Adachi, Masahiro Okuda

**Affiliations:** 1 Department of Anesthesiology, Aichi Gakuin University School of Dentistry, 2-11 Suemori-dori, Chikusaku, Nagoya, 464-8651 Japan; 2 Department of Anesthesiology, Nagoya University Graduate School of Medicine, 65 Tsurumaicho, Showaku, Nagoya, 466-8550 Japan

**Keywords:** Rocuronium bromide, Diluted administration, Onset time, Vascular pain

## Abstract

**Background:**

Rocuronium bromide (RB) is known to cause vascular pain. Although there have been a few reports that diluted administration causes less vascular pain, there have been no studies investigating diluted administration and the onset time of muscle relaxation. Therefore, we examined the influence of diluted administration of RB on the onset time of muscle relaxation and vascular pain.

**Methods:**

39 patients were randomly assigned to three groups: RB stock solution 10 mg/ml (Group 1), two-fold dilution 5 mg/ml (Group 2), or three-fold dilution 3.3 mg/ml (Group 3). After the largest vein of the forearm was secured, anesthesia was induced by propofol and 0.6 mg/kg of RB was administered. The evaluation method devised by Shevchenko et al. was used to evaluate the degree of vascular pain. The time from RB administration until the maximum blocking of T1 by TOF stimulation was measured.

**Results:**

There was no significant difference in escape behaviors of vascular pain among the three groups, and the onset time of muscle relaxation was significantly slower in Group 3 than in Group 1 (*p* = 0.033).

**Conclusion:**

Our results suggested that it is unnecessary to dilute RB before administration if a large vein in the forearm is used.

**Trial registration:**

UMINCTR Registration number UMIN000026737.

Registered 29 Mar 2017.

## Background

Rocuronium bromide (RB) is the most commonly used non-depolarizing neuromuscular blocking drug (NMBD) due to its shorter onset time and duration of action, and good operability as compared with other NMBDs. However, unconscious body response [1], which is an escape behavior from the pain caused upon RB administration in a single dose, is often observed. The degree of pain can sometimes be severe, causing a burning sensation [2], and it is reported that aspiration pneumonia may be caused by vomiting due to escape behaviors [3]. Many previous studies focused on drug administration to eliminate vascular pain before administration of RB, but there have been few studies indicating that diluted administration causes less vascular pain [4–7]. Furthermore, to the best of our knowledge, there have been no reports on diluted administration and the onset time of muscle relaxation. Therefore, we investigated the influence of diluted administration of RB on the onset time of muscle relaxation and vascular pain.

## Methods

### Ethics approval and consent to participate

This study was approved by the Ethics Committee of the School of Dentistry, Aichi Gakuin University (approval number: 490). Clinical trial registration was performed at UMIN-CTR before the start of the study (approval number: 000026737). After providing an adequate explanation regarding the aims of the research to all subjects, we obtained written informed consent from all the patients.

### Subjects

**Fig. 1:**
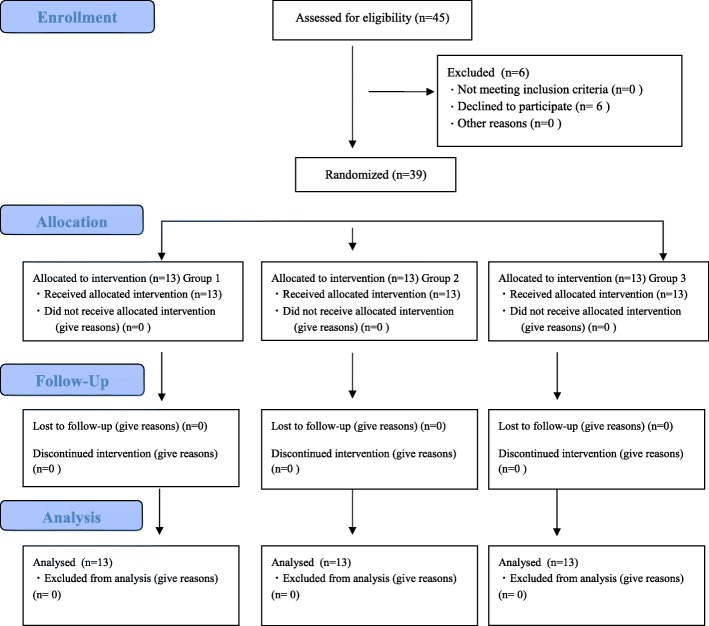
Consolidated Standards of Reporting Trials (CONSORT) recommended description for patient recruitment

Overall, 45 ASA-PS 1–2 patients between 20 and 70 years of age who were scheduled to undergo general anesthesia at our hospital. Patients who did not give consent, who had neuromuscular diseases, or who had a BMI ≥25 were excluded from the study. Patients were randomly assigned to the following three groups: patients receiving RB the stock solution of 10 mg/ml (Group 1:13 patients), patients receiving the two-fold dilution of 5 mg/ml (Group 2:13 patients), or patients receiving the three-fold dilution of 3.3 mg/ml (Group 3: 13 patients) (Fig. 1). To dilute RB, 0.9% saline was used.

### Methods of anesthesia

No premedication was used. After entering the operating room, the venous line was secured with a 20-G needle from the largest vein, excluding the cephalic vein, in the forearm. Furthermore, a TOF watch® was attached to the opposite arm. After induction of anesthesia with propofol at 1 to 2 mg/kg, it was visually confirmed that there was no residual propofol in the intravenous route, and RB at 0.6 mg/kg was administered in 10 s. The degree of vascular pain was evaluated based on the visual evaluation of escape behaviors from vascular pain. The time from RB administration until the maximum blocking of T1 by TOF stimulation was measured. In addition, the pH of the RB solution in Groups 1, 2, and 3 was measured using a pH meter (A&D AP-20).

### Evaluation parameters

For the patient background, sex, age, height, and weight were evaluated. We also investigated the onset time of muscle relaxation using TOF and the escape behaviors from the vascular pain. The degree of vascular pain was evaluated using the scale devised by Shevchenko et al. as follows: grade 1 = no response, grade 2 = movement at the wrist only, grade 3 = withdrawal involving the arm only (elbow or shoulder), grade 4 = generalized response or withdrawal in more than one extremity. Pain of grade 2 or above was considered to indicate vascular pain [1].

### Statistical analysis

The minimum sample size in total (30 patients) was calculated from a preliminary study based on the onset time of muscle relaxation (effect size 0.5, α-error level 0.05, and power 0.8).

The dropout rate in a preliminary study was 0.1. If an R dropout rate is expected, a simple but adequate adjustment is provided by N_d_ = N/(1-R)^2^ where N is sample size calculated assuming no dropout and N_d_ that required with dropouts [8]. Therefor our adjustment was 37.5 and 39 patients were randomly assigned to the three groups.

Statistical analysis was performed for age, height, weight, and the onset time of muscle relaxation using one-way ANOVA and multiple comparison by Tukey’s method. The chi-square test using the m × n division table was employed to investigate the impact of sex. In addition, escape behaviors from vascular pain were tested by the Kruskal-Willis test, and *p* < 0.05 was considered to indicate significance.

## Results

A CONSORT Diagram is shown in Fig. 1. Among 45 patients, six who refused to participate were excluded from the study, and the remaining 39 patients were randomly assigned to three groups. There were no patients who were unable to be followed up and 13 patients in each group were examined.

The patient backgrounds are shown in Table 1. There was no significant difference among the three groups regarding sex, age, height, or weight. Escape behaviors from vascular pain did not significantly differ among the three groups (Table 2). In addition, the onset time of muscle relaxion were 93.4 ± 28.1 s in Group 1, 101.7 ± 39 s in Group 2, and 136.1 ± 55.4 s in Group 3. There were significant difference between the Group 1 and Group 3 (*p* = 0.033) (Table 3).

**Table 1:**
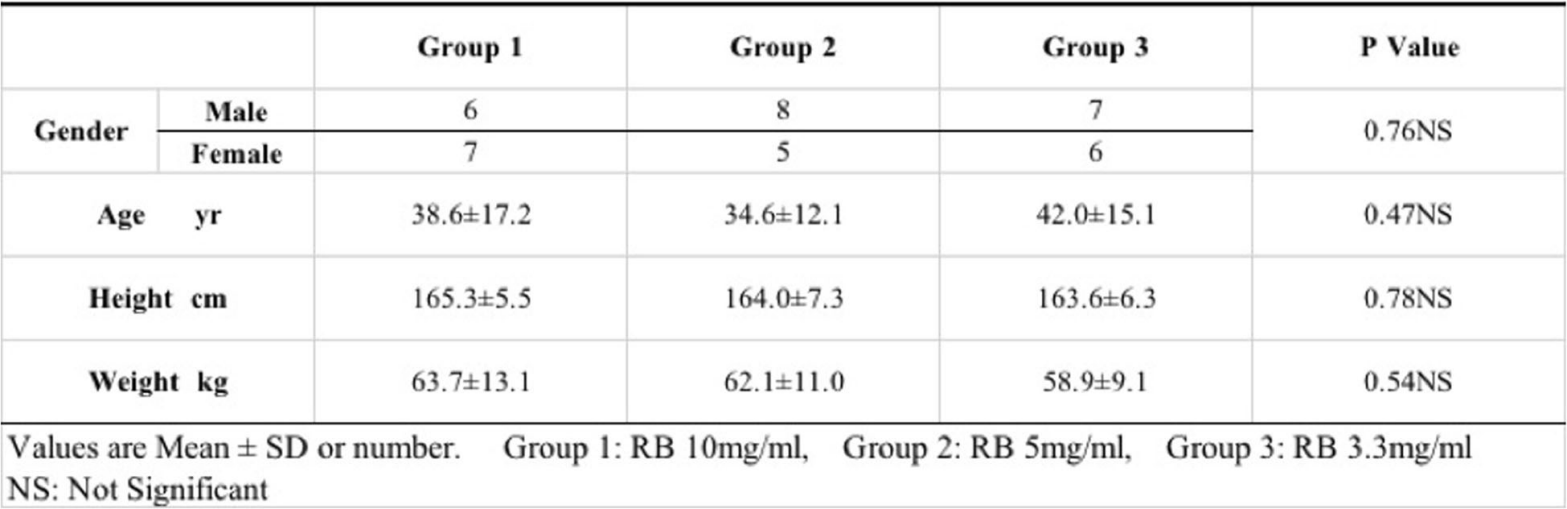
Characteristics of patients in this study

**Table 2:**
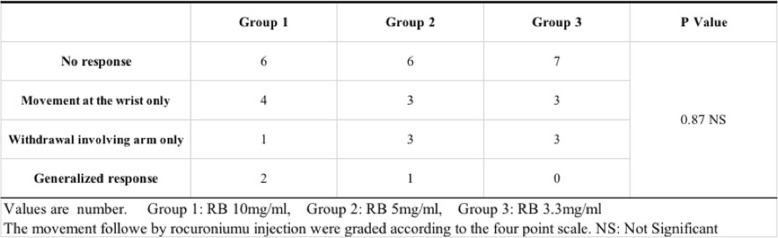
Grade of Withdrawal Movement Related to Rocuronium injection

**Table 3:**
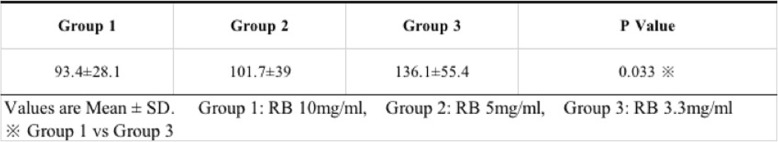
Onset Time of Neuromuscular Blockade

**Fig. 2:**
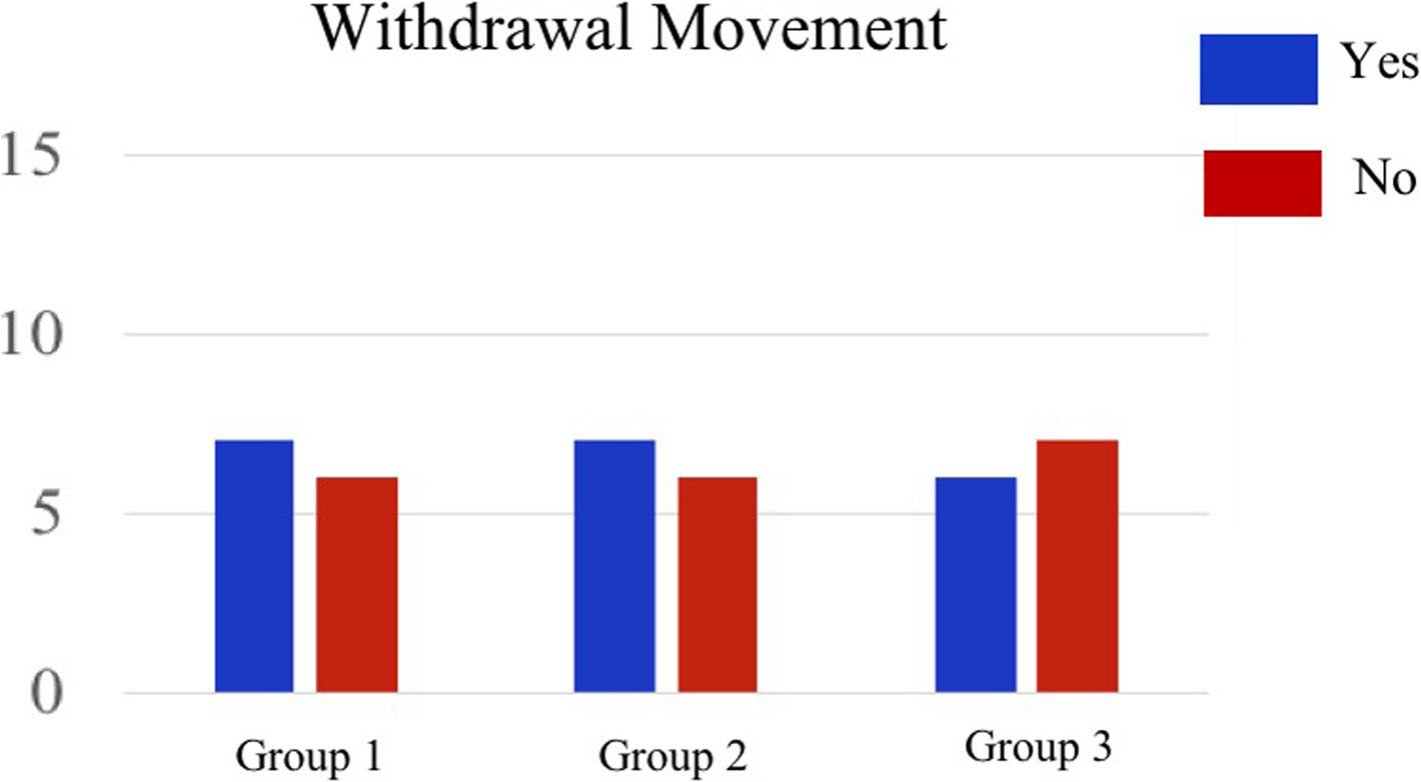
Overall incidence of withdrawal movement related to rocuronium injection. Group 1: RB 10 mg/ml, Group 2: RB 5 mg/ml, Group 3: RB 3.3 mg/ml. Values indicate the number of patients. There was no difference among the groups

Grade 2 or higher escape behaviors from vascular pain were observed in 53% (7/13) in both Group 1 and Group 2, and in 46% (6/13) in Group 3 (Fig. 2).

The pH of the RB solution was 4.0 for Group 1, 3.9 for Group 2, and 3.9 for Group 3.

## Discussion

In order for NMBD to induce muscle relaxation, it must bind to at least 70% of the nicotinic acetylcholine receptors. It has been reported that changes in the degree of binding of NMBD to the receptor may affect the time of onset of action [9], and that the nicotinic acetylcholine receptor consists of five units of adult-type α2βδε and fetal-type α2βδγ. The relationship between free molecules of NMBD that bind to the subunit and the time of onset of action has also been clarified [10]. In this study, RB was diluted with 0.9% physiological saline. The physiological saline used to dilute RB may have attenuated the affinity of RB to nicotinic acetylcholine receptors, and the change in free molecules due to dilution may have delayed the onset of muscle relaxation. In addition, as the onset time of NMBD and its titer are inversely correlated [9], it is necessary to increase the dose to accelerate the onset time of NMBD if the titer is low [11]. If the RB titer was decreased by dilution with 0.9% saline, the dose of diluted RB should be increased in order to accelerate the onset time of action. However, in this study, the dose of RB was fixed at 0.6 mg/kg in all three groups, which may explain why a difference was noted in the onset time of muscle relaxation between Group 1 and Group 3.

It has been reported that vascular pain due to single administration of RB is observed in approximately 50 to 80% of patients [12, 13]. Although the cause is not clear, the low pH and osmotic pressure of RB solution may stimulate chemical nociceptors on the vessel walls, and trigger the release of pain-inducing factors such as histamine and bradykinin [12, 14]. Regarding pH, it has been reported that RB solution with a pH of 4 does not cause vascular pain [12]. In this study, no difference was noted in vascular pain between Group 1 (RB solution pH 4.0) and Groups 2 and 3 (RB solution pH 3.9). Regarding osmotic pressure, Tuncali B et al. reported that the osmotic pressure of RB at 10 mg/ml is 308 mOsm/kg H_2_O and the osmotic pressure of RB at 1 mg/ml is 306 mOsm/kg H_2_O [6]; however, the osmotic pressure of RB at 10 mg/ml or 1 mg/ml did not cause vascular pain because these values were not different from the osmotic pressure of plasma (280–290 mOsm/kg H_2_O) [6]. Although we did not measure the osmotic pressure, that of RB at 5 mg/ml in Group 2 and RB at 3.3 mg/ml in Group 3 used in this study likely falls between the osmotic pressure of RB at 10 mg/ml of 308 mOsm/kg H_2_O and that of RB at 1 mg/ml of 306 mOsm/kg H_2_O. Taken together, the pH and osmotic pressure values of RB solution used are consistent with those reported in previous studies and are unlikely to affect vascular pain, which may be why there was no difference in escape behaviors from vascular pain noted in our study.

In our study, Group 2 and Group 3 did not exhibit differences in escape behaviors from vascular pain as compared with Group 1, which was inconsistent with previous studies [6, 7] demonstrating that diluted RB decreased vascular pain. When evaluating the reason for this different result, we focused on the position where the venous route was secured and vessel diameters. Similar to RB, propofol is known to cause vascular pain upon injection. Scott RP et al. demonstrated that administration of propofol through the median cubital vein can minimize pain due to decreased contact between the vessel wall and the drug [15]. In addition, Dalgleish DJ reported that RB administration through the median cubital vein did not cause vascular pain in 18 out of 20 patients [16]. This suggests that administrating RB through a larger vein can eliminate vascular pain due to less contact between the vessel wall and the drug. In the previous studies in which administration of diluted RB reduced vascular pain [6, 7], RB was injected through the dorsal digital veins in the hand. In contrast, we administered RB using the largest vein in the forearm. Thus, diluting RB is effective when administrating through relatively smaller veins but not when using a large vein, and no significant difference in escape behaviors from vascular pain is expected in such cases.

## Conclusion

We investigated the influence of diluted administration of RB on the onset time of muscle relaxation and vascular pain. We found that when diluted RB was administered through a large vein, there was no significant difference in escape behaviors from vascular pain. However, it should be noted that the onset time of muscle relaxation is delayed by dilution.

## Abbreviation

NMBD: Non-depolarizing neuromuscular blocking drug
RB: Rocuronium bromide

## References

[1] Shevchenko Y, Jocson JC, McRae VA, Stayer SA, Schwartz RE, Rehman M, Choudhry DK (1999). The use of lidocaine for preventing the withdrawal associated with the injection of rocuronium in children and adolescents. Anesth Analg.

[2] Moorthy SS, Dierdorf SF (1995). Pain on injection of rocuronium bromide. Anesth Analg.

[3] Lui JT, Huang SJ, Yang CY, Hsu JC, Lui PW (2002). Rocuronium-induced generalized spontaneous movements cause pulmonary aspiration. Chang Gung Med J.

[4] Memis D, Turan A, Karamanlioglu B, Sut N, Pamukçu Z (2002). The prevention of pain from injection of rocuronium by ondansetron, lidocaine, tramadol and fentanyl. Anesth Analg.

[5] Jung KT, Kim HJ, Bae HS, Lee HY, Kim SH (2014). So KY, LimKJ, Yu BS, Jung JD, an TH, park HC. Effects of lidocaine, ketamine, and remifentanil on withdrawal response of rocuronium. Korean J Anesthesiol.

[6] Tuncali B, Karci A, Tuncali BE, Mavioglu O, Olguner CG, Ayhan S, Elar Z (2004). Dilution of rocuronium to 0. 5 mg/mL with 0. 9% NaCl eliminates the pain during intravenous injection in awake patients. Anesth Analg.

[7] Shin YH, Kim CS, Lee JH, Sim WS, Ko JS, Cho HS, Jeong HY, Lee HW, Kim SH (2011). Dilution and slow injection reduces the incidence of rocuronium-induced withdrawal movements in children. Korean J Anesthesiol..

[8] Lachin JM (1981). Introduction to sample size determination and power analysis for clinical trials. Control Clin Trials.

[9] Kopman AF (1989). Pancuronium, gallamine, and d-tubocurarine compared: is speed of onset inversely related to drug potency?. Anesthesiology..

[10] Shear TD, Martyn JA (2009). Physiology and biology of neuromuscular transmission in health and disease. J Crit Care.

[11] Bhatt SB, Amann A, Nigrovic V (2007). Onset-potency relationship of nondepolarizing muscle relaxants: a reexamination using simulations. Can J Physiol Pharmacol.

[12] Borgeat A, Kwiatkowski D (1997). Spontaneous movements associated with rocuronium is pain on injection the cause?. Br J Anaesth.

[13] Lee YC, Jang YH, Kim JM, Lee SG (2009). Rapid injection of rocuronium reduces withdrawal movement on injection. J Clin Anesth.

[14] Lockey D, Coleman P (1995). Pain during injection of rocuronium bromide. Anaesthesia..

[15] Scott RPF, Saunders DA, Norman J (1988). Propofol: clinical strategies for preventing pain on injection. Anaesthesia..

[16] Dalgleish DJ (2000). Drugs which cause pain on intravenous injection. Anaesthesia..

